# Internal Medicine Enrichment and Development: A summer exploration program for pre-clerkship students

**DOI:** 10.36834/cmej.70070

**Published:** 2020-12-07

**Authors:** Alexandra Kobza, Kaitlin Endres, Shaima Kaka, Katina Zheng, Sarah Elias, Mimi Deng, Brandon Budhram, Aimee Li

**Affiliations:** 1 Faculty of Medicine, University of Ottawa, Ontario, Canada; 2 Department of Medicine, The Ottawa Hospital, Ontario, Canada

## Implication Statement

Medical students often have difficulty selecting a residency training program. The internal medicine clerkship rotation occurs primarily on the general internal medicine ward, making it difficult for students to experience the breadth of IM subspecialties prior to making career decisions. Herein, we describe a two-week student-led program (IMED: Internal Medicine Enrichment and Development) designed to give interested pre-clerkship students an overview of the internal medicine subspecialties in order to broaden their understanding of the opportunities within the field. We believe that medical students across the country would benefit from such exposure in order to make more informed decisions about residency.

## Énoncé des implications de la recherche

Les étudiants en médecine ont souvent de la difficulté à choisir un programme de résidence. Le stage de médecine interne (MI) se déroule principalement à l’étage, ce qui rend difficile pour les étudiants d’expérimenter l’étendue des sur-spécialités en MI avant de prendre des décisions relatives à leur carrière. Dans cet article, nous décrivons un programme de deux semaines dirigé par des étudiants (EDMI : Enrichissement et développement en médecine interne), conçu pour donner aux étudiants du pré-externat intéressés un aperçu des sous-spécialités en médecine interne dans le but d’élargir leur compréhension des opportunités offertes dans ce domaine. Nous croyons que les étudiants en médecine de partout au pays pourraient tirer profit d’une telle exposition pour leur permettre de prendre des décisions plus éclairées au sujet de leur résidence.

## Introduction

Medical students often find it challenging to select a residency program.^1^ A contributing factor may be that the residency application deadline often predates clinical exposure to all specialties. Researchers suggest most students decide on residency programs during pre-clerkship.^2^ However, without adequate exposure, this decision may be rooted in bias. Multiple studies support the notion that clinical exposures in pre-clerkship influence residency decisions, regardless of specialty.^3-4^

At many medical schools, the clerkship Internal Medicine (IM) rotation is based in the general IM ward.^5^ Therefore, it is difficult for students to experience the breadth of IM subspecialties prior to making career decisions. Two medical students conceived the idea for the IMED (Internal Medicine Enrichment and Development) program to provide students with adequate exposure to the breadth of IM subspecialties during pre-clerkship, and to allow for informed decisions regarding residency planning.

## Innovation

**Figure 1:**
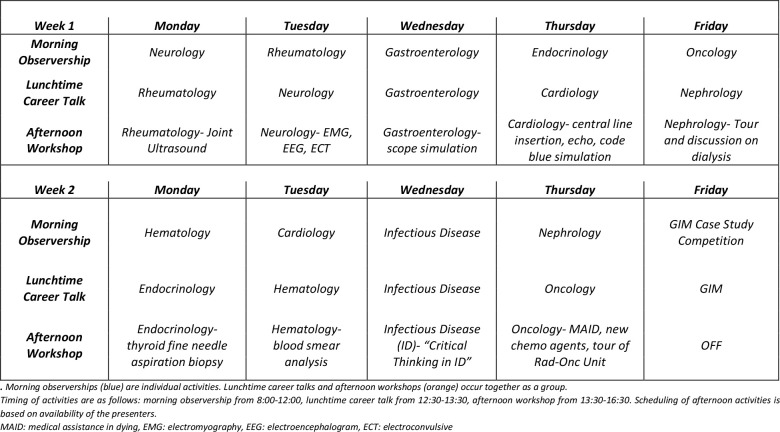
Sample schedule of a student participating in IMED

IMED is a two-week summer program organized by the medical student-run Internal Medicine Interest Group (IMIG) at the University of Ottawa. Participants spend each morning in a clinical setting observing a different IM subspecialty with one-on-one assignment to a staff physician. Participants convene at lunchtime for a career-oriented presentation delivered by a different subspecialty physician each day. Topics include scope-of-practice, work-life balance, and factors influencing choice of specialty. Students then proceed to hands-on workshops pertinent to said specialty, including procedural skills such as central line insertion, code blue simulation, and joint ultrasound. The final day of the program focuses on general IM and includes a team-based case competition that incorporates topics covered throughout the program. A sample student schedule is shown in Figure 1.

The IMIG executive committee garners support for the program by approaching department heads in all IM subspecialties to discuss program objectives. Each year interested departments with the staffing capacity to accommodate learners are included in the program. The number of interested departments determines the overall length of the program. Department heads recruit staff physicians to supervise student learners for morning observerships, and the department secretary schedules students in either hospital or community clinics. Department heads also nominate educators to deliver the lunchtime career talk and the afternoon workshop. The goal of the afternoon subspecialty workshop is to pique student interest by offering participants a unique insight into the chosen field, either by way of novel, innovative, and breakthrough practices and/or through an introduction to procedural skills not otherwise incorporated into the medical school curriculum. IMIG obtains funding for catered lunches and honorarium for presenters. Notably, the program can operate without funding if necessary, as students can bring personal lunches.

The organizing committee selected 20 interested pre-clerkship students (from first and second year, where each class consists of 167 students) via random lottery for the inaugural IMED in 2018. The organizing committee reviews the program size annually depending on the number of students that interested subspecialties can accommodate. Students’ interest in IM residency is not a pre-requisite to participate.

## Outcomes

The REB exempt this study as a program evaluation. The sixteen participants of IMED in 2019 completed pre-program and post-program surveys (survey completion was 100%). To assess whether students felt they had been exposed to the breadth of IM through IMED, they responded to 3 statements using a 5-point Likert scale (strongly disagree, disagree, neutral, agree, strongly agree) before and after IMED:

- I understand what a career in IM entails in terms of hours, pay, research opportunities, job prospects, and lifestyle
- I understand which subspecialties of IM are more “procedure heavy” vs. “medicine heavy”
- I understand the differences between community and academic practice in IM

A Wilcoxon Signed-ranks test revealed that post-IMED scores were significantly higher than pre-IMED scores with Z=3.5, Z=3.4, and Z=3.2, all *p* < 0.01 for each question (I-III) respectively. Following IMED, participants were asked if they were more, less or equally likely to pursue internal medicine as a career. Thirteen participants (13/16, 81%) indicated that IMED made them more likely to pursue a career in IM, two (13%) answered less likely, and one (6%) answered equally likely. Our results suggest that IMED provided students with new information regarding a career in IM, which will likely aid in making career decisions. Due to the self-selected sample and the prospective nature of the questions, it is possible that results may be influenced by projection bias and confirmation bias.

## Next steps

In future iterations of IMED, we plan to include other common subspecialties such as respirology and geriatrics, as well as smaller subspecialties where there exists a societal demand, such as addiction and pain medicine.^6^ We encourage other universities to consider implementing such a program to give medical students the opportunity to explore the breadth of IM at an early stage. Organizing such an initiative requires student dedication to recruit subspecialty departments and to coordinate the efforts of faculty to accommodate student learning. In the future, we hope to follow participants longitudinally to examine IMED’s influence on residency selection. Adding a control group comprised of interested applicants not selected for the program would allow us to determine whether the program promotes informed decision-making surrounding residency and career planning.
